# Advances in Duchenne Muscular Dystrophy: Diagnostic Techniques and Dystrophin Domain Insights

**DOI:** 10.3390/ijms26083579

**Published:** 2025-04-10

**Authors:** Julija Sarvutiene, Arunas Ramanavicius, Simonas Ramanavicius, Urte Prentice

**Affiliations:** 1 State Research Institute Center for Physical Sciences and Technology (FTMC), Sauletekio Av. 3, LT-10257 Vilnius, Lithuania; julija.sarvutiene@ftmc.lt (J.S.); arunas.ramanavicius@chf.vu.lt (A.R.); simonas.ramanavicius@ftmc.lt (S.R.); 2 Department of Physical Chemistry, Institute of Chemistry, Faculty of Chemistry and Geosciences, Vilnius University, Naugarduko St. 24, LT-03225 Vilnius, Lithuania; 3 Department of Personalised Medicine, State Research Institute Center for Innovative Medicine, Santariskiu St. 5, LT-08410 Vilnius, Lithuania

**Keywords:** Duchenne muscular dystrophy, gene mutation, rare diseases, dystrophin

## Abstract

Abnormalities in X chromosomes, either numerical or structural, cause X-linked disorders, such as Duchenne muscular dystrophy (DMD). Recent molecular and cytogenetic techniques can help identify DMD gene mutations. The accurate diagnosis of Duchenne is crucial, directly impacting patient treatment management, genetics, and the establishment of effective prevention strategies. This review provides an overview of X chromosomal disorders affecting Duchenne and discusses how mutations in Dystrophin domains can impact detection accuracy. Firstly, the efficiency and use of cytogenetic and molecular techniques for the genetic diagnosis of Duchenne disease have, thus, become increasingly important. Secondly, artificial intelligence (AI) will be instrumental in developing future therapies by enabling the aggregation and synthesis of extensive and heterogeneous datasets, thereby elucidating underlying molecular mechanisms. However, despite advances in diagnostic technology, understanding the role of Dystrophin in Duchenne disease remains a challenge. Therefore, this review aims to synthesize this complex information to significantly advance the understanding of DMD and how it could affect patient care.

## 1. Introduction

In 1861, the French neurologist Guillaume-Benjamin-Amand Duchenne published pioneering research in the Archives Générales de Médecine. He provided the first detailed clinical description of a progressive muscle-wasting disease affecting young boys. His work introduced innovative diagnostic techniques, significantly advancing neuromuscular disease research [1]. Duchenne muscular dystrophy (DMD or Duchenne) is a severe and lethal form of dystrophinopathy [2]. DMD affects about 1 in every 5000 to 6000 live male [3] births globally. Caused by mutations in the DMD gene, dystrophin (protein crucial for muscle stability), DMD causes permanent damage to muscle fibers and their replacement with fatty and fibrous tissue. Symptoms that can be seen usually appear in early childhood, when affected individuals experience difficulty with walking, climbing or descending stairs, experience frequent falls, and difficulty getting up. Over time, muscle tissue deteriorates, muscles weaken, and atrophy. By adolescence, wheelchairs are required in many cases, and as the disease progresses, respiratory and heart muscles are affected, leading to life-threatening complications. Despite advances in care, DMD remains a devastating and incurable disease that has a significant impact on both patients and their families. Individuals with DMD experience failure in respiratory and cardiac muscles [4], ongoing muscle damage [5,6], widespread inflammation [7], and the significant absence or low expression of the Dystrophin protein [8,9]. This leads to a loss of muscle function [10] due to a weak connection between the actin cytoskeleton and connective tissue [11], as well as an increased risk of acute myocardial infarction [12] and cardiomyopathy, which can be fatal [13]. The Dystrophin gene, which expresses the Dystrophin protein, stands as the longest gene in the human genome, spanning 2.2–2.6 million DNA base pairs and consisting of 79 exons [14]. Dystrophin domains are crucial components of the Dystrophin protein, playing a vital role in maintaining muscle’s structural integrity [15]. This protein is essential in connecting the cytoskeleton of muscle fibers to the extracellular matrix, providing structural support during muscle contractions. Our efforts are directed towards the development of therapeutic interventions for muscular dystrophy. The various domains of the Dystrophin protein collaborate to maintain muscle cell structure and assist the protein in interacting with other proteins in the Dystrophin Glycoprotein Complex (DGC). The Dystrophin domains are essential for maintaining muscle cell structure and function. Understanding its role is crucial for developing treatments for muscular dystrophy despite the challenges posed by gene mutations.

## 2. Structure and Function of the Dystrophin

### 2.1. Overview of the Dystrophin Isoforms

The X-linked DMD gene that causes Duchenne [16,17] mutation is located on the X chromosome in the Xp21 region [18,19]. This occurs due to disruptions in replication, transcription, RNA processing, translation, and protein processing [18,19]. Additionally, Dystrophin protein isoforms are different forms of protein encoded by different exons of the DMD gene through alternative splicing [20]. Moreover, genes have two types of segments: exons and introns. Introns are unnecessary to produce a fully functioning protein, although exons are required.

Research on the longest isoform, Dystrophin Dp427, has led to significant breakthroughs. It is fascinating to note that the brain has a high level of Dystrophin expression [21], surpassed only by muscle. However, the role of Dystrophin in the brain is still unclear [22]. Although small in the genome, this gene has garnered global attention due to its potential to improve human health. Dystrophin protein makes up only 0.002% of the total protein in muscle cells [23]. It is crucial for the integrity of the cell membrane [24] and is necessary to maintain muscle structure [25] and strength [26,27]. As detailed in Table 1, Dystrophin isoforms allow the DMD gene to produce specific protein forms tailored to different body parts and functions. This mechanism enables various tissues to use the same gene while generating slightly different proteins to fulfill their requirements [28]. Recent research by Wijekoon et al. [29] has found that mutations in the DMD gene can affect the expression of shorter dystrophin isoforms (Dp140, Dp71, and Dp116), leading to impaired motor functions in individuals with DMD. The loss of these isoforms also influences the progression of the disease. Understanding the function and regulation of dystrophin protein isoforms can provide valuable insights into muscle and tissue health and identify potential therapeutic targets for conditions caused by Dystrophin deficiencies [30,31].

According to recent research conducted by De Feraudy et al. [32], the level of Dystrophin present may significantly impact the severity of their DMD symptoms. This study supports the idea that the amount of Dystrophin protein can influence the characteristics of patients with DMD mutations, including those with DMD and BMD (Becker muscular dystrophy) [33]. The patients were divided into three groups based on their Dystrophin levels (the total number of patients included was 90): group A, with no Dystrophin; group B, with 0–5% Dystrophin; and group C, with more than or equal to 5% Dystrophin. The study assessed the age at which the first symptoms appeared, loss of mobility, drop in vital capacity and left ventricular ejection fraction, interventions such as spinal fusion, tracheostomy, non-invasive ventilation, and mortality across the three groups. Group A, which has no Dystrophin, exhibited severe phenotypes across all the items, and most of the patients in this group were diagnosed with DMD. On the other hand, the population diagnosed with BMD was primarily distributed between groups B (0–5%) and C (≥5%), which showed a milder phenotype than group A. Based on this, the level of Dystrophin in BMD could potentially be a criterion for improving symptoms in DMD. However, Dystrophin levels in BMD are pretty variable, reported as 10–90% and 3–78% [34] of the healthy population. Beekman et al. [35] showed that the concentration of Dystrophin in individuals with BMD was lower than that determined for healthy people (31 individuals) and higher than that determined for DMD (17 individuals). However, the lowest level of Dystrophin in BMD was similar to that in DMD. As a result, the BMD population with high Dystrophin levels was combined with the healthy population with low Dystrophin levels.

In 1986, the first fragments of DMD gene cDNA were identified, followed by the prediction of the 3685 amino acid protein sequence and the production of antibodies specific to the Dystrophin protein in 1987 [36]. This invention allowed researchers to understand DMD genetics and molecular pathology better. The discovery of the Dystrophin protein led to extensive research on myofiber membrane cytoskeleton, membrane repair, muscle regeneration, and regeneration failure. This eventually led to the development of molecular therapeutics and detection based on understanding Dystrophin protein structure and function. This historical perspective highlights the events related to the initial identification of the Dystrophin protein. However, there were a total of 12,109 deaths attributed to DMD during the study period. Out of these, heart failure contributed to 2419 (20%) deaths, while respiratory failure contributed to 3763 (31%) [37].

**Table 1:** Dystrophin isoforms and their functions.

Dystrophin Isoform	Size and Length	Expression	Function
Dp427 [38,39]	~427 kDa, 3685 amino acids.	Skeletal, cardiac muscle, the plasma membrane of muscle cells, and the brain. It plays a vital role in preserving the structural integrity of muscle cell membranes.	Maintaining the cytoskeleton of muscle cells and their connection to the extracellular matrix provides stability during muscle movement and contributes to brain function.
Dp260 [40,41]	~260 kDa, 2150 amino acids.	Retina.	Maintains the structural and functional integrity of the retina by preserving the connection between photoreceptor cells and the retinal pigment epithelium.
Dp140 [42,43]	~140 kDa, 1260 amino acids.	Brain and fetal tissues have less pronounced expression in skeletal muscle and are absent in the retina. Transcription of Dp140 begins upstream from exon 45.	Dp140’s exact function is less defined than other Dystrophin isoforms, but it is believed to contribute to cognitive functions and brain development in the central nervous system.
Dp116 [44]	~116 kDa, 1061 amino acids.	Schwann cells which are peripheral nervous systems.	Dp116 is important for the peripheral nervous system and Schwann cell integrity. It helps with cytoskeleton organization and cell membrane stability and provides mechanical support for muscle fibers during contraction and relaxation. Although not enough for normal muscle function on its own, its presence is critical for overall muscle health [45,46].
Dp71 [47]	~71 kDa, 620 amino acids.	Various tissues include the brain, kidney, liver, lung, retina, and cardiac muscles. Transcription of Dp71 begins between exon 62 and exon 63.	Dp71 maintains cell membrane integrity in non-muscle tissues, is involved in synaptic function in the brain, and helps keep the structural integrity of retinal cells.
Dp40 [48]	~40 kDa, 365 amino acids.	The expression pattern of Dp40 is not as well characterized as other Dystrophin isoforms, but it is known to be present in various tissues, including the brain.	This isoform lacks the entire β-dystroglycan binding site and the C-terminal domain. Dp40 is involved in signal transduction and maintaining cell structure. It localizes neurons to synaptic vesicles and interacts with presynaptic proteins. The exact role of Dystrophin in the brain is still unclear despite high expression levels in the brain and muscles.

### 2.2. Overview of the Dystrophin Domains

**Figure 1:**
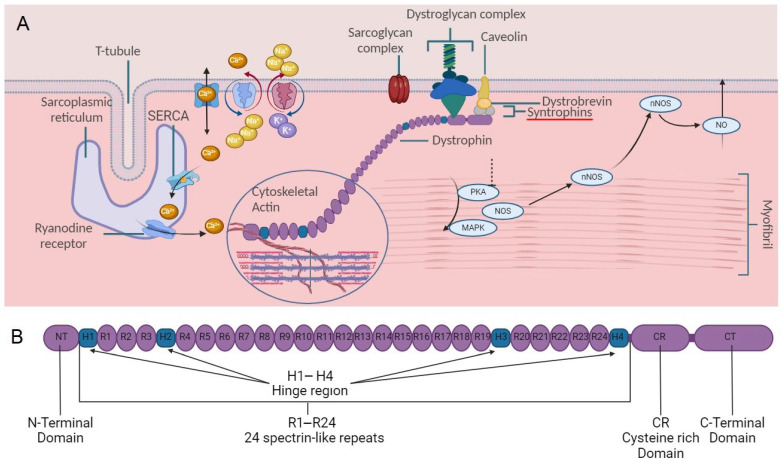
(**A**) The presence of Dystrophin protein in cells ensures the balance of ions and other cellular functions. This is achieved by the mechanical connection between the Dystrophin-associated protein complex (DAPC), which comprises dystroglycan and sarcoglycan complexes, and the cytoskeletal actin. This connection further links to mitogen-activated protein kinase (MAPK) and protein kinase A (PKA) pathways and nitric oxide synthase (NOS) signaling, allowing proper localization of neuronal NOS (nNOS) and resulting in the release of vasodilative nitric oxide (NO) [38]. (**B**) Schematic of Dystrophin (Dp427)—24 spectrin-repeats, four hinges, N-terminal (NT), cysteine-rich (CR), and C-terminal (CT) domains.

While Dystrophin is expressed in many tissues of the body, it plays a crucial role in stabilizing the muscle membrane (sarcolemma) [49] by connecting the actin cytoskeleton to the extracellular matrix during muscle contraction. Dystrophin is a cytoplasmic protein essential for binding to actin filaments, as it serves as a critical signaling molecule for numerous muscle fiber functions [50]. Based on an analysis of the amino acid sequence, Koening and his colleagues [26] predicted that the Dystrophin molecule could be divided into four distinct domains [36] (Figure 1B).

In the N-terminal (NT) actin-binding (N-ABD or ABD, 1–246) domain [51,52], the Dystrophin protein interacts with the cytoskeleton and shares similarities with α-actinin and β-spectrin. Mutations such as Leu54→Arg, Ala168→Asp, Ala171→Pro, and Tyr231→Asn [53] destabilize the protein’s function by destabilizing the domain fold rather than substituting essential residues for actin binding [54].

**Figure 2:**
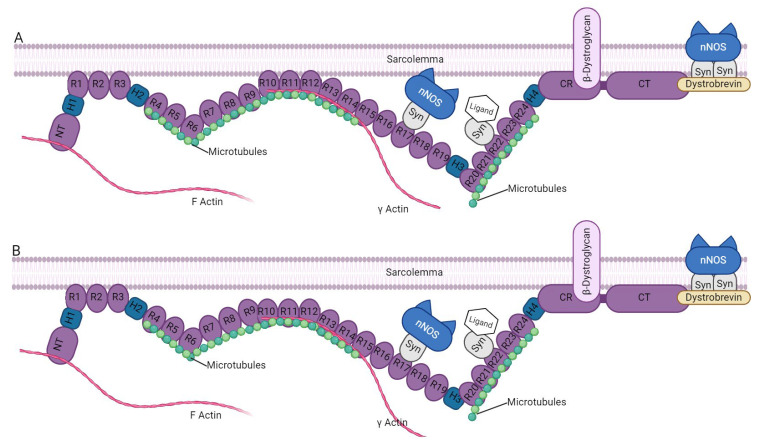
Model for the dual requirement of spectrin-like repeat R17 of Dystrophin and α-syntrophin for nNOSμ localization to the sarcolemma. In muscle (**A**), Dystrophin binds to the sarcolemma through four independent membrane-binding subdomains (R1–R3; R10–R12; CR; and CT regions). However, Dystrophin binds to the sarcolemma in the heart (**B**) through three independent membrane-binding domains (R1–R3; CR; and areas of CT). We have highlighted the key partners that play an essential role in binding, such as microtubules, nNOS, and the sarcolemma.

The central-rod triple-helical [55] domain of Dystrophin is a flexible spacer (24 spectrin-like repeats (R) and four hinge regions (247–3045 residues containing a second ABD (ABD2) between actin-binding and membrane-associated domains [56,57]. It is believed to play a role in dimerization, essential for the protein’s functioning [58]. The dimerization [59] occurs through the binding of one monomer [60,61] to another, forming a dumbbell-shaped tetramer system’s structure [62]. The research by Rybakova et al. [63,64] discovered Dystrophin’s ability to interact with actin filaments within residues 1416–1880. This study revealed that Dystrophin’s amino terminus and central rod domain can bind to actin filaments, providing stabilization against actin depolymerization [65]. Additionally, the central rod of Dystrophin interacts differently with sarcolemma phospholipids [66] in cardiac and skeletal tissue [63,67], possibly due to distinct spectrin-like properties (Figure 2). The central rod of Dystrophin anchors it to the sarcolemma through hydrophobic and electrostatic interactions with phosphatidylserine [65]. This observation may provide a partial explanation for the targeting of Dystrophin to the sarcolemma, and it reinforces the idea that the rod domain likely plays a much more significant and complex role in Dystrophin function than previously thought. Interestingly, the central rod of Dystrophin interacts differently with sarcolemma phospholipids in cardiac and skeletal tissue, possibly due to distinct spectrin-like properties [66]. In cardiomyocytes, the spectrin repeats R1–R3, C-terminal, and cysteine-rich (CT/CR) domains, which are crucial for binding to the sarcolemma. However, binding to the skeletal muscle sarcolemma also occurs via R10–R12 [68]. The γ-actin cytoskeleton is linked to spectrin repeat regions R11–R17 of ABD, which contain basic amino acid residues. Dystrophin’s rod domain directly interacts with microtubules [69], and disruptions in this connection can worsen DMD pathology. The molecule’s central domain has several partners, including neuronal nitric oxide synthase (nNOS) [70], filamentous actin, and membrane lipids [71]. The rod region is divided into three sub-regions by four predicted hinges, providing additional flexibility [72]. The α-helical structure of the repeats was confirmed experimentally, and the folding is observed when the repeat extends some residues into the adjoining sequence repeat by the Gratzer group [73]. Ankyrin-B is necessary to prevent microtubule loss [74,75,76]. Subdomain R1–R3 binds exclusively to the skeletal muscle membrane and prefers the intercalated disc in the heart, while subdomain R10–R12 only partially localizes to the membrane in skeletal muscle. Studies [77,78,79] have shown that large deletions of Dystrophin can still produce functional proteins, although not fully functional. Large deletions within the rod domain can still produce partly functional proteins.

The C-terminal (CT) domain that has protein-binding sites (CT, 3361–3685 residues) interacts directly with β-dystroglycan and indirectly with other members of the Dystrophin-glycoprotein complex (DGC) [80]. Dystrophin links the cell’s interior and exterior by connecting to the dystroglycan complex (DGC). The deletion of one protein of the DGC renders the entire myocardium dysfunctional. Each component of the DGC relies on the presence and function of others.

A cysteine-rich (CR) domain containing 14 cysteines that have a sequence (CR, 3080–3360 amino acid residues) similar to the C-terminus of a-actinin and has two incomplete Ca^2+^ binding motifs [81]. The CR domain is a highly conserved region that binds with α-dystrobrevin and α1-, β1-syntrophins [82], and stabilizes the sarcolemma. With 420 amino acids and numerous cysteines, it anchors to the sarcolemma via ankyrin-B [83]. Mutagenesis studies [68] have shown that the CR domain in Dystrophin is crucial for creating a binding site for the transmembrane protein β-dystroglycan—recent research by De Feraudy et al. [32] indicated that Dystrophin levels can significantly impact the severity of DMD. Beekman et al. [35] found that the concentration of Dystrophin in BMD was lower than in healthy individuals and higher than in DMD patients. This suggests that Dystrophin levels in BMD could potentially predict symptom severity in DMD.

Zhao et al.’s [61] study was conducted to evaluate the subcellular localizations of different subdomains of Dystrophin in vivo. The well-known CR domain was studied along with several highly conserved MBDs of Dystrophin that can independently interact with the sarcolemma. The newly identified MBDs were R1–R3, R10–R12, and CT. It was found that the CT subdomain binds to the sarcolemma in both skeletal and cardiac muscle, and further, it restores the DGC. In the same study, A. Zhao et al. [61] showed that subdomain R1–R3 exhibited exclusive membrane binding in skeletal muscle and preferred the intercalated disk in the heart. However, subdomain R10–R12 only showed partial membrane localization in skeletal muscle.

## 3. Dystrophin Detection and Regulation

### 3.1. Dystrophin Detection Issues and Perspectives

Dystrophin stabilizes muscle fibers by connecting the cytoskeleton and extracellular matrix via amino and carboxy terminals [84]. Mutations in DMD can lead to truncated or non-functional Dystrophin protein expression [85]. Three types of DMD gene changes can cause the loss of [86] Dystrophin: point mutations in approximately 20% of DMD patients [87], duplications, and deletions, which are the most common types of DNA change in people with DMD, almost 70% of DMD patients [88,89]. The early and accurate diagnosis of DMD is crucial for managing the disease effectively. Genetic testing [90,91,92,93], including Dystrophin gene deletion and duplication detection, is commonly used to screen for DMD. Large deletions and duplications of genetic material are not randomly distributed but instead tend to cluster in regions of exons 2–20 and 45–53. These regions correspond to the NT domain and the beginning and end of the helical repeat region, respectively. Point mutations comprise the remaining third of mutations and are more evenly distributed along the gene. However, a few of them are also found in the NT domain. A greater understanding of Dystrophin function and its role in muscles could improve our knowledge of the pathogenesis of DMD.

Aartsma-Rus et al. found that individuals with more than 5% Dystrophin expression, as evaluated by immune assays and normalized by relative dysferlin, tend to have milder phenotypes compared to those with undetectable Dystrophin levels [2,94]. However, the lowest concentration of Dystrophin in DMD patients is only 5-fold higher than that in the lowest limit of quantification (LLOQ) [95,96], which is not detectable by immunoassay technology combining electrochemiluminescence and multiarray technology for detecting multiple proteins in a sample and Western blotting due to their low sensitivity. Furthermore, the spike recovery rate in DMD samples was 107%, indicating that this assay can accurately and confidently evaluate increases in Dystrophin levels in DMD patients.

Diagnosing DMD through molecular testing is challenging due to the gene’s large size, complexity, and high mutation rate. Spontaneous mutations account for about 30% of cases, while point mutations, gross deletions, and duplications cause the remaining cases [86]. Numerous detection methods have been reported to improve high-selectivity detection technologies that can accurately and quantitatively detect genetic changes, variations, or mutations in the Dystrophin protein or genes. These methods include next-generation sequencing [97,98], fundamental [99,100,101,102], molecular [103,104,105,106], antibody-based immunoassays [107,108,109,110,111], and enzyme-based immunoassays [112,113,114,115], optical/colorimetric [116,117], fluorescent [118,119,120,121,122,123], and optical detection [124,125,126,127,128] methods are of particular interest among the new methodologies. These methods rely on hybridizing target DNA and a modified substrate with tags such as radioactive, hazardous chemicals [129], fluorescent [130,131], chemiluminescent, or based on application nano [132,133,134,135,136,137,138,139,140] or magnetic [141] particles with specific properties [142,143,144,145,146]. Furthermore, the comprehensive analysis requires sequencing all 79 exons and eight promoters [147,148]. Mass spectrometry [95,149,150], Denaturing High-Performance Liquid Chromatography (DHPLC) [151], and sequencing offers significant advantages for Dystrophin detection, including high sensitivity, specificity, detailed protein characterization, and quantitative analysis. However, it also comes with disadvantages such as its time-consuming aspect, complex sample preparation (protein extraction, digestion, and purification steps that can introduce methods variability), high costs, technical expertise requirements, limited throughput, and challenges associated with the analysis of large, complex proteins [152]. Despite the challenges, these methods continue to hold significant value in Dystrophin research and clinical analysis, particularly when there is a need for detailed protein-level information. ELISA [153,154,155,156,157,158,159], Western blotting [158,160,161,162], and immunoassays [163,164,165,166] are highly time-consuming processes that demand expensive instrumentation and specialized labor. Moreover, antibodies [143] are characterized by high production costs and exhibit low stability at elevated temperatures. Furthermore, the chemical modification of antibodies poses significant challenges, rendering them a less optimal choice as a biosensor recognition element.

Different mutations can affect detection accuracy depending on the specific exon or region within the DMD gene where the mutation occurs. Mutations in DMD protein lead to a shortened but functional Dystrophin protein, particularly in the central rod domain between exons 10 and 60 [167,168]. For example, certain exons are more likely to have deletions or duplications, and detection methods may be more effective for these regions. Mutations in less common regions may be more challenging [169,170] if the methods are not specifically tailored to those areas. It is generally easier to detect large deletions or duplications using MLPA (Multiplex Ligation-dependent Probe Amplification) [171] or comparative genomic hybridization (CGH) [121,122,123,172,173]. On the other hand, pinpoint mutations or small insertions/deletions [174] may necessitate more sensitive methods such as Sanger sequencing or next-generation sequencing (NGS), as several research [175] groups have recently developed screening methods to detect exonic sequence variations, followed by only direct sequence analysis of variant fragments. Mutations in the DMD gene are often associated with significantly elevated serum CK (Creatine kinase) levels. However, elevated CK levels, which indicate [176] muscle damage, are not specific to DMD. They can also be elevated in other muscle disorders, injuries, or after strenuous exercise. Serum CK levels are a valuable screening test for Dystrophin-related conditions in symptomatic patients. NGS has transformed the diagnosis of dystrophinopathies by providing a high-throughput, cost-effective, and accurate method for detecting deletions, duplications, and point mutations in the DMD gene. It can identify a wide range of mutations, including small insertions and deletions (indels) as well as deep intronic variants, and is faster and more comprehensive than traditional Multiplex Ligation-dependent Probe Amplification (MLPA). However, a limitation of NGS is that it can generate variants of uncertain significance (VUS), which necessitates further validation. Additionally, it has a limited capacity to detect large genomic rearrangements or mosaicism in low-frequency populations.

Jung D et al. [78] developed an in vitro binding assay to study the β-dystroglycan and Dystrophin interaction sites. They used Dystrophin CT portion fusion proteins to investigate the interaction. The results showed that a minimum β-dystroglycan binding motif is confined to amino acids 3054–3271, which include exons 62–67. However, additional amino acids located downstream between residues 3271 and 3446 are required for maximum binding. The binding motif comprises several amino acids distributed throughout the second half of hinge four and the CR domain. The results showed that Dystrophin and syntrophin cosediment with the GST-β-DGct-agarose beads but not with the GST-agarose beads. This suggests that either Dystrophin or syntrophin can bind the cytoplasmic domain of β-dystroglycan. The binding of Dystrophin and syntrophin to the GST-β-DGct-agarose beads is likely due to the β-dystroglycan–Dystrophin interaction rather than the β-dystroglycan–syntrophin interaction. These findings suggest that Dystrophin can bind to both syntrophin and β-dystroglycan through different binding sites. Analitical methods have been developed for detecting dystrophin, each offering unique benefits and limitations depending on the application context, as shown in Table 2.

**Table 2:** Dystrophin detection methods advantages and disadvantages.

Method	Advantages	Disadvantages	References
Multiplex Ligation-dependent Probe Amplification (MLPA)	- The gold standard for detecting large deletions/duplications in the DMD gene. - Cost-effective and widely available. - ~1% variant frequency DNA (blood and muscle biopsy).	- Cannot detect small mutations (e.g., point mutations and small indels). - May miss complex structural variations.	[177,178]
Next-Generation Sequencing (NGS)/Whole-Exome Sequencing (WES)	- Detects a wide range of mutations (point mutations, small indels, and deep intronic variants). - Suitable for comprehensive mutation screening and carrier detection. - It is more sensitive than MLPA. - ~0.1–1% variant frequency DNA (blood, saliva, and muscle biopsy).	- Variants of uncertain significance (VUS) require further validation. - May not efficiently detect large structural rearrangements or mosaic mutations.	[179,180]
Whole-Genome Sequencing (WGS)	- Provides complete genetic information, including deep intronic mutations and regulatory regions. - Useful for research and discovery of novel mutations. - ~0.1% variant frequency DNA (blood, saliva, and muscle biopsy).	- High cost and significant data analysis burden. - Not yet a routine clinical test.	[181,182]
Optical Genome Mapping (OGM)	- Detects significant structural variations (e.g., inversions, translocations, and duplications) that NGS may miss. - Higher resolution than traditional karyotyping or FISH. - ~5 kb rearrangements DNA (blood and fibroblasts)	- It is less effective in detecting single nucleotide variants (SNVs). - Requires specialized equipment and expertise.	[183,184]
CRISPR-Based Detection Methods	- High specificity for known mutations. - Potential for rapid point-of-care testing in the future. - ~1 fM (mutation-specific) DNA (blood and saliva)	- Currently in the research phase and not yet widely available. - Requires further validation for clinical use.	[185,186]
RNA Sequencing (RNA-seq)	- Identifies cryptic splicing mutations missed by DNA sequencing. - Provides insights into dystrophin gene expression and exon-skipping therapy targets. - ~1 TPM (transcripts per million) RNA (muscle biopsy)	- It requires a muscle biopsy, which is invasive. - RNA quality and degradation can affect results.	[187,188,189]
Immunohistochemistry (IHC)/Western blot	- Directly assesses dystrophin protein expression and distribution. - Useful for confirming molecular diagnoses. - ~10 ng of dystrophin protein (muscle biopsy)	- It requires a muscle biopsy, which makes it invasive. - Limited ability to detect genetic causes of the disease.	[10,33,190]

Improved detection methods have made it possible to identify many specific mutations within the Dystrophin gene that cause DMD. Large deletions and duplications of genetic material are not randomly distributed but instead tend to cluster in regions of exons 2–20 and 45–53. These regions correspond to the NT domain with helical repeat regions, respectively. Point mutations comprise the remaining third of mutations and are more evenly distributed along the gene. However, a few of them are also found in the NT domain. Increased knowledge of Dystrophin’s function and its role in muscles has led to a better understanding of DMD.

### 3.2. Upregulation of Dystrophin

The primary biochemical function of Dystrophin in skeletal muscle myofibers [191] is to enhance the stability of the plasma membrane and protect it from damage caused by physical force. Although Dystrophin has no signaling or enzymatic functions, it connects with multiple other proteins with such functions, such as neuronal nitric oxide synthase (nNOS). Mutations (whether frameshift or nonsense) lead to different degrees of deficiency of Dystrophin protein isoforms. If the mutation occurs in 29 exons [192], it may inactivate Dystrophin isoforms expressed in the Purkinje cells, brain, and muscle while leaving other isoforms like Dp260 (found in the retina), Dp140 (brain), Dp116 (peripheral nerve), and Dp71 (ubiquitous) intact [193]. Mutations occurring in all 17 exons, as referenced by Bucher et al. (2019) and Taylor et al. (2010), have the potential to result in a deficiency of all Dystrophin isoforms across various tissues [194,195]. The clinical findings clearly indicate that the absence of Dp260 in the retina results in the loss of night vision and alterations in electroretinography [196]. This phenotype correlates with the location of the DMD gene mutation and the predicted Dp260 isoform. This could suggest that mutations in the N-terminal of the gene may result in the loss of more Dystrophin isoforms, leading to more severe cognitive involvement and developmental brain abnormalities. Numerous attempts have been made [85,197,198] to find treatments to restore Dystrophin. These treatments include gene therapy, stop codon read-through therapy, and exon skipping.

Adeno-associated virus (AAV) vectors are a popular choice for delivering therapeutic genes due to their ability to transduce a variety of cell types, including muscle cells, and their relatively low immunogenicity [199,200,201,202] but is accompanied by significant challenges, including immune responses that impact the safety and efficacy of these treatments. Here is an in-depth look at the outcomes and challenges observed in human trials, focusing on generating autoantibodies and other immune-related issues. In the context of DMD, AAV vectors are engineered to carry the genetic code for micro-dystrophins, which are shortened versions of the dystrophin protein (approximately 150 kDa as opposed to the normal 427 kDa). These micro-dystrophins retain the essential functional domains needed to restore muscle function but are small enough to be packaged into AAV vectors. Clinical trials [203] have evaluated AAV vectors encoding three slightly different micro-dystrophin variations. Micro-dystrophin induces transgene expression, and it is well tolerated, with only mild adverse events [204,205,206,207]. Clinical trials have shown that AAV vectors encoding micro-dystrophins can effectively induce transgene expression and are generally well tolerated, with only mild adverse events, such as temporary immune responses. There have been promising improvements in dystrophin production and muscle function, although long-term efficacy and durability are still under investigation. Importantly, the systemic delivery of rAAVrh74.MHCK7 micro-dystrophin has demonstrated minimal adverse effects, indicating its safety. This vector has been associated with increased micro-dystrophin expression in skeletal muscle biopsies. Functional improvements, including better motor outcomes, were observed in some patients, particularly younger cohorts. Studies have also shown that once micro-dystrophin is expressed, it remains stable in muscle cells, which contributes to improved muscle integrity and reduced fibrosis over time.

However, studies have identified potential risks associated with excessive micro-dystrophin expression in the heart, which may lead to dilated cardiomyopathy and heart failure due to competition with native utrophin and protein degradation pathway overload. These findings highlight the importance of optimizing expression levels and monitoring cardiac health in future applications [203,208]. Pre-existing neutralizing antibodies (NAbs) against AAV vectors are common, limiting eligible patients for clinical trials. Those with high NAb titers are often excluded. Introducing micro-dystrophin can trigger an immune response in patients with little or no native dystrophin, leading to autoantibodies that can neutralize the treatment or cause chronic inflammation. Solid Biosciences has reported serious adverse events, including immune-mediated thrombocytopenia and complement activation, resulting in temporary trial halts. The overexpression of micro-dystrophin in cardiac tissue may also lead to adverse outcomes, like dilated cardiomyopathy, possibly due to competition with endogenous utrophin or protein degradation overload [209,210,211]. In addition to neutralizing antibodies, T cell-mediated immune responses can occur after AAV administration. These responses may target transduced cells expressing the AAV capsid proteins, leading to inflammation and loss of transgene expression. Cytotoxic T lymphocytes (CTLs) can recognize AAV-derived peptides presented on the surface of infected cells and attack those cells, thus impairing the efficacy of the therapy. This immune attack on transduced cells could result in a transient or permanent loss of the therapeutic dystrophin protein expression [210,211].

Stop codon read-through therapy is a treatment that targets nonsense mutations in the Dystrophin mRNA [212]. These mutations introduce premature stop codons, causing the production of truncated and non-functional Dystrophin proteins. SiRNAs (small interfering RNAs) are short RNAs consisting of 20 to 25 nucleotides that can affect gene expression [213] by targeting specific gene fragments for activation or silencing. Additionally, siRNA can control or regulate dystrophin gene transcription by suppressing or reducing the amount of dystrophin mRNA. By reducing the amount of dystrophin mRNA, protein synthesis and its quantity can be controlled. SiRNAs interact with the RNA-induced silencing complex (RISC) [214], and when the antisense strand of the siRNA binds to the target mRNA, the RISC cleaves the mRNA. However, delivering siRNAs to specific tissues, such as muscles, is challenging. Unmodified siRNAs have a short half-life and are quickly eliminated from the body. Chemical modifications [215,216] and conjugations to the siRNA are necessary to avoid degradation and clearance and improve transport to target tissues. The therapy promotes the suppression of these stop codons, allowing the synthesis of full-length Dystrophin proteins, which can function properly. The main goal of stop codon read-through therapies is to produce intact Dystrophin proteins. The read-through drug is Ataluren (3-[5-(2-fluorophenyl)-[1,2,4]oxa-diazol-3-yl]-benzoic acid; C_15_H_9_FN_2_O_3_), a compound formerly called PTC124. This treatment brings hope to 13% [179,217,218] of DMD patients with nonsense mutations.

Exon skipping therapy [219] is a treatment that uses U7 snRNPs or oligonucleotides (AONs) to express an antisense sequence to modify pre-mRNA splicing. AONs are designed to hide specific exons from the RNA splicing machinery from shorter and semi-functional Dystrophin proteins. Exon skipping aims to restore the reading frame [220] of the Dystrophin gene by skipping over specific exons with mutations. This process enables the production of a shortened Dystrophin protein. Exon skipping offers a potential treatment for around 80% of all DMD mutations [221], encompassing deletions and duplications. The most frequently deleted exons in DMD patients are exons 51, 53, and 45, mutated in approximately 20%, 13%, and 12% of patients, respectively [222,223]. The region between exons 45 to 55 in DMD is a hotspot where mutations tend to accumulate. Exon 51 skipping applies to 14% of patients, representing the largest DMD population who may benefit from single exon skipping [224]. Alternatively, genome editing can be used to achieve exon deletions.

Utrophin, a homolog of dystrophin, is naturally expressed during embryonic development and in specific adult tissues, such as neuromuscular junctions. It compensates for dystrophin by interacting with the same components of the dystrophin-associated protein complex (DAPC), providing structural stability to the sarcolemma. Like dystrophin, the utrophin gene is too large (~13 kb) to be efficiently delivered using adeno-associated virus (AAV) vectors, which have a cargo capacity of ~4.7 kb. This limitation has led to research on mini- and micro-utrophin constructs. While truncated versions of utrophin have been shown to restore some function, they may not fully replicate the mechanical or signaling roles of the full-length protein. These constructs’ long-term safety and efficacy remain to be thoroughly evaluated in vivo. Therapies to upregulate utrophin [225] expression may provide a functional substitute for Dystrophin in individuals with DMD. It can provide some structural support to muscle fibers and partially compensate for their absence. Research has shown that both full-length and truncated forms of utrophin can help prevent muscle degeneration [226,227,228] and improve pathology associated with [225] dystrophin deficiency in animal models. Higher levels of full-length utrophin synthesis have been associated with better muscle function. Hence, boosting utrophin expression could result [228] in more substantial therapeutic advantages for DMD patients in a dosage-dependent manner. Unlike other therapeutic methods, utrophin modulation [226] appears non-toxic in preclinical studies, suggesting a favorable safety profile for clinical use.

CRISPR/Cas9 applications [229,230,231] have shown promise in treating DMD, including correcting point mutations, deleting exons, reframing, knock-ins, and base editing. Long et al. [232] used CRISPR/Cas9 to correct the DMD gene sequence in mdx embryos, resulting in genetically mosaic animals. Duchene et al. [233] demonstrated the restoration of the dystrophin open reading frame (ORF) and functional dystrophin synthesis using SaCas9 in a humanized mouse model of DMD. More giant insertions can also be achieved with Pickar-Oliver et al. [234] achieving full-length dystrophin in mice through homology-independent target integration. The following research by Dhoke et al. outlines a CRISPR-Cas9 gene editing method to introduce mini-DYS cDNA for any DMD mutation downstream of exon 44, connecting exon 44 to exon 58 [235]. The study demonstrates the expression of mini-DYS in terminally differentiated myotubes from two gene-corrected DMD iPSC lines in vitro. Additionally, it shows the in vivo rescue of DYS expression after transplanting myogenic progenitors from both gene-edited DMD iPSC lines. Generally, base editing methods have corrected nonsense mutations, and prime editing has restored dystrophin in cardiomyocytes. However, challenges include limited protein stability and segmental repair of the sarcolemma. Retreatment may be necessary due to muscle damage and potential loss of therapeutically altered genetic material. Off-target mutations and immune responses to Cas9 and rAAV vectors also pose risks, but strategies to mitigate these challenges are being explored. CRISPR-Cas9 technology has recently been adapted for diagnostic purposes, enabling the targeted amplification and detection of dystrophin gene mutations with high specificity. This approach offers advantages such as greater mutation specificity compared to PCR-based methods and the potential for point-of-care applications. However, it is still in the early stages of research and is not yet widely available in clinical settings. Further validation is needed to ensure its diagnostic robustness.

Dystrophin restoration therapies have shown immense promise in treating muscular dystrophy. However, their efficacy is limited to intact muscle tissue [236]. To address this limitation, Stop Codon Read-Through and Exon Skipping therapies have been developed, which focus on dystrophin transcripts produced exclusively in skeletal muscles. The micro-dystrophin cDNAs, controlled by muscle-specific promoters, are transcribed only in skeletal muscles and the heart.

Strategies to enhance dystroglycan function include improving alpha-dystroglycan glycosylation to restore its binding affinity for extracellular matrix (ECM) components like laminin. The therapeutic modulation of glycosylation pathways, using small molecules or gene therapies targeting enzymes, has shown potential in preclinical models. Enhancing dystroglycan’s interaction with utrophin or engineered dystrophin constructs could partially compensate for dystrophin loss. Emerging research on dystroglycanopathies is also providing insights into novel gene and protein engineering approaches to optimize dystroglycan functionality, offering a complementary pathway to address DMD-associated muscle pathology [237].

Protein-upregulating Nucleic Acid-Based Therapies (NBTs) were developed but faced multiple challenges [238,239,240]. There is a need to understand better the genetic determinants and pathophysiology of diseases, to rationally design drug molecules, to conduct advanced studies on the pharmacokinetic and pharmacodynamic characteristics of drugs, to develop more convenient modes of drug administration, and to establish specific regulatory procedures. Despite the challenges encountered in the protein-upregulation field, the experience of companies working to improve NBT technology has demonstrated that advances lead to better treatment outcomes. Improvements in gene sequencing techniques and the newfound hope inspired by the feasibility of NBTs in genetic disorders will expand genetic studies. More rare disorders and subpopulations of ’common’ diseases will be attributed to specific genetic defects. Given that many disease-associated mutations lead to target protein insufficiency, the applicability of protein-upregulating NBTs will increase.

### 3.3. Autophagy and Its Role in DMD

Autophagy is essential for maintaining skeletal muscle health by facilitating the removal of damaged mitochondria and protein aggregates. This process helps to prevent cellular stress and ensures muscle integrity. When autophagy is dysregulated, it significantly contributes to the development of DMD, a condition marked by muscle degradation, inflammation, and impaired regeneration [241]. Targeting autophagy, either directly or through its upstream regulators, presents a promising strategy for improving outcomes in DMD. Future research should aim to optimize these therapeutic approaches for clinical use and to investigate the complex interactions between autophagy, inflammation, and mitochondrial dysfunction in DMD [242]. Addressing autophagy could lead to a multifaceted treatment strategy that complements the existing dystrophin-targeted therapies, potentially enhancing the management of this challenging condition. A reduction in autophagy hampers the clearance of defective mitochondria, increasing oxidative stress and contributing to mitochondrial dysfunction. Additionally, impaired autophagy can worsen inflammatory responses and activate fibroblasts, thereby promoting tissue fibrosis. Several signaling pathways are involved in the regulation of autophagy in muscle; among these, the mechanistic target of rapamycin complex 1 (mTORC1) is known to negatively regulate autophagy by inhibiting ULK1, a kinase necessary for initiating autophagosome formation [243]. In DMD, the chronic hyperactivation of mTORC1 suppresses autophagy, resulting in protein buildup and muscle degeneration. Therapeutic strategies such as rapamycin treatment have shown promise in reactivating autophagy and mitigating muscle damage. Furthermore, FOXO proteins are known to positively regulate autophagy and muscle regeneration, while DEAF1, a downstream target of FOXO, plays a pivotal role in regulating the genes associated with autophagy. Enhancing the activity of FOXO or increasing DEAF1 expression may promote autophagy-mediated muscle repair in DMD [244,245]. DMD is also characterized by impaired mitochondrial dynamics, as reduced mitophagy leads to the excessive production of reactive oxygen species (ROS), which aggravates oxidative stress and myofiber damage. Improving mitophagy through pharmacological or genetic interventions has demonstrated potential in enhancing mitochondrial quality control and decreasing oxidative stress in preclinical models of DMD.

### 3.4. Muscle Mass Research Aspects

Research on muscle mass in the context of DMD has recently focused on several critical aspects. Myostatin, the transforming growth factor-beta (TGF-β) superfamily [246], is primarily produced and secreted by skeletal muscle cells. It acts as a negative regulator of muscle growth, inhibiting muscle growth. Myostatin inhibitors [247], including antibodies or small-molecule compounds, hinder myostatin’s activity. By preventing myostatin from receptors on muscle cells, these inhibitors effectively neutralize its inhibitory effects on muscle growth. The inhibition of myostatin leads to the promotion of increased muscle hypertrophy or muscle growth. In conditions characterized by muscle wasting, such as DMD, myostatin inhibition holds promise as a therapeutic approach. Muscle weakness and degeneration in DMD result from the absence of dystrophin protein, and myostatin inhibition aims to compensate for this loss by promoting muscle growth and regeneration. This results in improvements in muscle strength and function. However, excessive muscle growth resulting from myostatin inhibition may cause potential side effects such as muscle fibrosis, insulin resistance, and cardiac hypertrophy. However, therapies that target myostatin, such as myostatin inhibitors or antibodies, have exhibited promise in preclinical studies and early-stage clinical trials for DMD. These therapies aim to block myostatin signaling to promote muscle hypertrophy, which can improve muscle function in DMD patients. The NF-κB signaling pathway is important in muscle inflammation and atrophy in DMD. Preclinical studies [248,249] have demonstrated that targeting NF-κB signaling can reduce muscle damage and improve muscle strength. Fibrosis, characterized by the excessive deposition of scar tissue muscles, is a hallmark of DMD and contributes to muscle wasting. Several therapies have been explored to reduce fibrosis and promote muscle regeneration. These include agents that target the TGF-β pathway, which plays a central role in fibrosis, and drugs that modulate the activity of fibroblasts and other cells involved in tissue repair. Gene therapy using viral vectors to deliver functional copies of the dystrophin gene has also shown promise in preclinical studies and early-phase clinical trials. Muscle stem cells [250], satellite cells, can potentially regenerate damaged muscle tissue in DMD. Therefore, therapies that aim to enhance satellite cells or transplant healthy muscle stem cells into affected muscles have been explored as potential treatments for DMD.

In conclusion, therapeutic advancements in DMD muscle mass research focus on targeting critical pathways involved in muscle growth, regeneration, and fibrosis. These approaches aim to preserve muscle mass, ultimately slowing the progression of muscle degeneration. Recent therapeutic advancements in muscle mass research for DMD show great promise in enhancing well-being and prolonging the lives of affected individuals.

### 3.5. Artificial Intelligence (AI) Integration in Duchenne Detection

Recent advancements in omics technologies have generated substantial data for disease modeling. These challenges aggregating and interpreting diverse data types such as transcriptomic, epigenomic, functional, and textual data from separated databases. Integrating Artificial Intelligence (AI) [251] into detecting and managing Duchene is a relatively recent development. However, AI applications in medical diagnostics, including those for DMD, have gained significant traction mainly over the past decade. Many artificial intelligence (AI) approaches to muscle ultrasound image analysis have limitations in real-time clinical applications in neuromuscular medicine because of their computational demands and non-standardized testing methods. Traditionally, muscle ultrasound images or videos have been used to identify whether muscles are normal or pathological visually; however, unlike needle electromyography, they cannot distinguish between neurogenic and myogenic origins. Visual evaluations [252] can detect apparent abnormalities, such as changes in brightness, atrophy, tumors, or abnormal movements; however, they require substantial expertise and achieve only approximately 70 % efficacy in differentiating between healthy and abnormal muscle tissues.

Real-time texture analysis using RFC (random forest classifier) is an effective and efficient method for diagnosing neuromuscular disorders using muscle ultrasound. The published Noda Y et al. [253] method has significantly reduced the computational demands associated with artificial intelligence methods, making it practical for clinical use. Validation of this model in additional patients through video evaluations has highlighted its practical utility in the clinical setting. Performing real-time texture analysis without disrupting clinical workflows is a significant advance over traditional diagnostic methods. Integrating quantitative analysis directly into the clinical trial process further increases diagnostic accuracy and significantly accelerates decision-making, improving patient outcomes and clinical efficiency. Other studies [254,255,256,257] have investigated the application of deep learning models to analyze MRI scans to identify early biomarkers of Duchenne. These publications emphasized the utilization of artificial intelligence algorithms to process muscle MRI imaging, demonstrating promising and successful outcomes in detecting early indicators of muscle degeneration and fatty infiltration, which are hallmarks of DMD [258].

Furthermore, research has utilized machine learning techniques to interpret genomic data and identify mutations in the dystrophin gene. Herzog et al. [259] paper discussed the application of predictive analytics to clinical data, including patient medical records, biometrics, and laboratory results, to forecast the progression of DMD. The models developed in the study by Vera C et al. [260] offered valuable insights into disease progression patterns and contributed to personalizing patient treatment plans. While AI has demonstrated exceptional success in disease diagnosis, particularly through image analysis. However, its applications extend beyond imaging to areas such as blood biomarker analysis, drug discovery, and modeling disease progression.

AI-driven algorithms have revolutionized the analysis of blood-based biomarkers by enhancing diagnostic accuracy and predicting disease risk. Machine learning models can analyze multi-omics data—including proteomics, metabolomics, and genomics—to detect disease signatures with unprecedented sensitivity and specificity. Rajkomar et al. [261,262] demonstrated that deep learning models could predict sepsis in ICU patients up to 48 h before clinical manifestation, outperforming traditional scoring systems.

AI has been employed to analyze circulating tumor DNA (ctDNA) and other blood-based biomarkers, enabling the early detection of cancers such as lung and pancreatic cancer with over 90% accuracy in some models [241,263]. AI-enhanced algorithms have been used to diagnose conditions like diabetes and cardiovascular diseases by evaluating patterns in blood chemistry, achieving up to 96% accuracy in predicting diabetic complications.

AI accelerates drug discovery by predicting molecular interactions, optimizing compound selection, and identifying novel therapeutic targets. Recent advances in deep learning have enabled the development of AI-driven platforms for high-throughput screening and de novo drug design [264,265]. AI models can analyze longitudinal patient data to forecast disease progression, aiding in personalized treatment strategies. For instance, deep learning models have been applied to predict Alzheimer’s disease progression from multimodal patient data [266,267].

## 4. Conclusions

Duchenne muscular dystrophy is a genetic disorder that causes the absence of Dystrophin, usually due to mutations in the DMD gene, which presents unique challenges for diagnosis. Traditional diagnostic approaches relied heavily on muscle biopsies and immunohistochemistry, but recent advancements have improved accuracy, reduced invasiveness, and enhanced mutation characterization. However, in some instances, mutations are partially corrected, resulting in Dystrophin in specific muscle fibers. This occurrence can provide crucial insights into the correlation between Dystrophin levels and the severity of DMD in patients. An accurate assay to measure Dystrophin is vital for evaluating the progress of DMD. In addition, such an assay determines the normal range of Dystrophin in healthy populations. This information could help establish a standardized goal for Dystrophin levels in a clinical setting.

Conditional approval has been granted for read-through and exon-skipping drugs, but vector-mediated gene therapy holds promise pending clinical usage approval. However, minor changes in dystrophin production or functional improvements reported so far detect clinical effects during relatively short-term trials. Given the difficulties in conducting control population studies for DMD, optimizing study designs or drug structures may aid in evaluating efficacy. Real-world data analysis could provide insights into long-term drug efficacy, including survival improvements. Recent therapeutic approaches for restoring dystrophin in DMD patients show promise, but their efficacy remains uncertain. Challenges in generating evidence include the rarity of DMD, difficulty in enrolling enough patients for well-designed studies, and lack of standardized outcome measures. Preclinical issues include improving the efficiency of exon skipping and vector-mediated gene therapy, optimizing micro-dystrophin potency, minimizing immunological risks, and ensuring therapy durability. Cell therapy faces limitations such as limited cell availability, low survival and migration rates, potential tumor formation, and immune responses. Addressing these challenges is crucial for advancing DMD therapy.

## Data Availability

Data sharing does not apply to this article as no datasets were generated.

## Abbreviations

The following abbreviations are used in this manuscript:

ABD	Actin-Binding Domain
BMD	Becker muscular dystrophy
CR	cysteine-rich
cDNA	Complementary DNA
CT	C-terminal
DAPC	Dystrophin-Associated Protein Complex
DGC	Dystrophin Glycoprotein Complex
DMD	Duchenne muscular dystrophy
DNA	Deoxyribonucleic Acid
MAPK	Mitogen-Activated Protein Kinase
nNOS	neuronal nitric oxide synthase
NO	nitric oxide
NT	N-terminal
PKA	protein kinase A
RNA	Ribonucleic Acid
